# The ethics and economics of organoid commercialization: potential donors’ perspectives

**DOI:** 10.1186/s12910-025-01269-3

**Published:** 2025-07-29

**Authors:** Hanna de Groot, Manon van Daal, Regina W. Hofland, Inez Bronsveld, Karin R. Jongsma, Renske M.T. ten Ham

**Affiliations:** 1 Julius Center for Health Sciences and Primary Care, Department of Epidemiology & Health economics, University Medical Center Utrecht, Utrecht University, Utrecht, The Netherlands; 2 Julius Center for Health Sciences and Primary Care, Department of Bioethics & Health Humanities, University Medical Center Utrecht, Utrecht University, Utrecht, The Netherlands; 3 Department of Pulmonology, University Medical Center Utrecht, Utrecht University, Utrecht, The Netherlands

**Keywords:** Donor perspectives, Commercialization, Organoids, Ethics, Economics

## Abstract

Advancing organoid technology requires human tissue donations and collaboration between researchers and commercial parties. However, many potential donors have reservations about commercial involvement in organoid research. To better understand these reservations, we conducted four focus groups with potential donors. Two focus groups were held with individuals with cystic fibrosis (*n* = 10). One focus group included individuals with neurodegenerative diseases (Parkinson’s or Huntington’s disease) (*n* = 4) and the other consisted of individuals with neurological disease (epilepsy) (*n* = 5). Four themes were identified: (1) benefits and concerns regarding commercial involvement, (2) trust in involved parties in research, (3) control over commercial parties and (4) appreciation of donors. To address these themes, we recommend that researchers and commercial parties communicate transparently and effectively, actively engage and appreciate donors, implement robust oversight mechanisms and (re)establish trust and trustworthiness through responsible practices. These considerations can help researchers and commercial parties work toward responsible and sustainable organoid research.

**Supplementary Information:**

The online version contains supplementary material available at 10.1186/s12910-025-01269-3.

## Background

Organoids are three-dimensional, self-organizing cell structures that can be grown in vitro from human stem cells [1]. These miniature organ-like structures can model a range of functional and diseased tissues, allowing researchers to study mechanisms of (human) development and disease [2]. In addition, organoids can be used for developing and testing innovative treatment approaches, screening drugs for personalized medicine, and hold promise for applications in clinical transplantation [3].

Because of the range of potential applications and promising initial results of organoid technology, public and private partners are increasingly interested in using human-derived organoids [4]. For instance, human intestinal and brain organoid models have been developed and used to study Cystic Fibrosis (CF), brain diseases and neurological disorders [5–8]. While intestinal organoids have advanced to clinical applications in drug development and drug screening for CF, brain organoids are currently primarily used to study disease mechanisms [5–8].

Research with human-derived organoids is paired with ethical and economic implications [4, 9]. Prior studies indicated that potential donors have reservations about commercial involvement in organoid research, yet the underlying reason for this attitude remains unclear [10–15]. Some donors have expressed concerns about the ethical aspects of profiting from donated human tissues [12, 14] and capitalizing on the illnesses of individuals [11, 14]. Given that commercial parties are essential to realize and accelerate drug development by providing investment, expertise and infrastructure, further scrutiny is warranted [16].

Since organoid research necessitates human donors, understanding potential donors’ perspectives on commercial involvement is imperative. Therefore, we aim to identify and assess potential donors’ views on commercial involvement in organoid research. To investigate the perspectives of potential donors, we selected two organoid models as reference cases: intestinal and brain organoid models. We selected these models based on their distinct applications in different (pre-clinical and clinical) research phases and their differences in ethical sensitivity. For example, the current debate surrounds the potential moral status of brain organoids, given the possibility of consciousness [17–19]. This difference in ethical sensitivity could be of influence on how commercialization of both organoid models is perceived. These two cases will allow us to compare and analyse different perspectives depending on the disease background or tissue origin.

This paper presents the results of a focus group study into the perspectives of potential donors on commercial involvement in organoids. By building on the findings of prior empirical studies [11, 14], this paper aims to further clarify the underlying reasons for potential donors’ reservations regarding commercial involvement and propose suggestions for responsible commercialization in organoid research.

## Methods

### Study design

In this focus group study, we investigated potential donor perspectives on commercial involvement in research with organoids. The term ‘potential donor’ refers to individuals who are not necessarily asked to donate at this time but potentially are relevant candidates for future organoid research based on their medical background. We define commercial involvement as either collaboration between commercial and academic parties or independent research by commercial parties using organoids from public or commercial biobanks. Focus groups are a qualitative research method to gather a comprehensive understanding of attitudes, opinions, and experiences within a specific context [20]. The interaction among participants is used to obtain broader and deeper information than is possible in an individual interview. To introduce complex concepts, clarify the roles of various stakeholders in organoid research and familiarize respondents with the topic, we developed vignettes. Vignettes are short descriptive scenarios illustrating specific situations, designed to invite responses from participants [21]. Vignettes can make topics more relatable and encourage participants to engage with the topic by envisioning the scenarios [21].

### Participant selection and recruitment

The participants of this study ​​were divided into two groups based on their potential relevant organoid model (intestinal or brain). Two focus groups were conducted for each group. Participant selection focused on Dutch-speaking adult individuals whose condition made them potentially eligible for biospecimen donation for organoid research. For the intestinal organoid focus groups, individuals with CF were selected (Table 1). For the brain organoid focus groups, one focus group included individuals with neurodegenerative diseases (Parkinson’s or Huntington’s disease) and the other consisted of individuals with neurological disease (epilepsy) (Table 1).

Adults with CF were recruited via healthcare professionals from the University Medical Center Utrecht (IB, RH and Evelien C. van der Hout), located in Utrecht, the Netherlands, and the Dutch patient organization for CF (*Nederlandse Cystic Fibrosis Stichting)*, via their social media channels such as Facebook and newsletters. Individuals with epilepsy, Parkinson’s and Huntington’s disease were recruited by the Dutch patient organizations for epilepsy, Parkinson’s and Huntington’s disease (*Epilepsie NL*,* Parkinson Vereniging and Vereniging van Huntington*) via their websites and newsletters. Participant recruitment took place between February 2024 and April 2024.

**Table 1:** Overview of respondent groups and respondent characteristics

	Focus group 1 (*n* = 4)	Focus group 2 (*n* = 6)	Focus group 3 (*n* = 5)	Focus group 4 (*n* = 4)
**Group type**	Intestinal organoid	Intestinal organoid	Brain organoid	Brain organoid
**Diagnosis**	Cystic fibrosis (4)	Cystic fibrosis (6)	Epilepsy (5)	Parkinson (3)
				Huntington (1)
**Gender**				
Man	3	3	2	2
Woman		3	3	2
Other	1			
**Age**	33–59	28–46	20–55	41–69

### Vignettes and topic list

To prepare for the focus groups, explorative interviews were conducted to get insight into potential routes of commercial involvement in organoid research and product development. Interviewed stakeholders included academic organoid researchers (*n* = 2), and employees from both public (*n* = 2) and commercial biobanks (*n* = 2). Insights from these interviews were used to create vignettes (in Microsoft PowerPoint) that illustrated two evolving scenarios of potential commercial involvement in research with organoids for drug development. These scenarios illustrate real-world examples of commercial involvement in the Netherlands and Europe.

In all focus groups, participants were asked to envision donating a biospecimen for organoid development. The type of biospecimen and organoid model varied per group. Participants in the intestinal organoid group envisioned donating an intestinal biopsy to develop intestinal organoids, while those in the brain organoid group were asked to envision donating blood samples to develop brain organoids.

Participants were presented with two scenarios. In the first, participants were asked to envision donating their biospecimen for organoid research via a public biobank. In this scenario, academic researchers collaborated with commercial parties in conducting research and drug development. Building on this scenario, three parameters were varied sequentially: (1) the nature of collaboration between academic and commercial parties (leading or collaborating role), (2) whether compensation was offered (yes or no), and (3) whether study outcomes were made publicly available (yes or no).

In the second scenario, participants were asked to envision donating their biospecimen for organoid development to a commercial biobank. Here, the commercial party purchased organoids from a commercial biobank and conducted the research without further collaboration with an academic party. Building on this scenario, the parameters compensation (yes or no) and public availability of study results (yes or no) were also varied sequentially. The scenarios and their variations were presented in the same order across all focus groups.

The vignettes were complemented with a topic list developed for this study, containing open-ended questions about expectations, potential benefits and risks and the conditions for acceptance of organoid commercialization (appendix 1). The vignettes and topic list were piloted by MvD and HdG in collaboration with a junior researcher, who had no prior knowledge on organoids or this study, to assess their accessibility, comprehensiveness and flow. The vignettes and topic list allowed participants to discuss issues they considered relevant, whilst also ensuring some consistency in the topics that were discussed across focus groups. If necessary, the moderator asked follow-up questions to gain a deeper understanding of the arguments brought up by the respondents.

### Data collection

Four focus groups were conducted between May 2024 and August 2024. The focus groups lasted between 60 and 90 min. All focus groups took place online via MS Teams because of feasibility and health safety reasons. The focus groups were conducted in Dutch. Two focus groups were moderated by one researcher (MvD) and observed by another researcher (HdG). In the other focus groups, the roles were reversed. The focus groups were recorded, transcribed verbatim and pseudonymized.

### Data analysis

Data from this study was analyzed thematically using the constant comparative method, which involves going back and forth from the data to develop codes, concepts, and themes [22]. Full transcripts were independently coded by two researchers (HdG and MvD) using NVivo 15 software (release 15.0.0). Next, codes were checked for consistency between the researchers (HdG and MvD) and interpretations and suitability were critically discussed and adjusted accordingly. After consensus, codes were iteratively developed into higher-order themes and discussed amongst all authors. Per theme, results were analyzed descriptively. The aim was to explore the range of perspectives shared during the focus groups rather than to quantify the percentage or number of participants expressing a certain opinion. Representative quotes were chosen to illustrate the patients’ experiences and translated ​into English by HdG. Each quote was accompanied by an identifier (e.g. R1.2 refers to respondent 2 in focus group 1).

## Results

The analysis of the focus groups resulted in four themes: (1) benefits and concerns regarding commercial involvement, (2) trust in involved parties in research, (3) control over the use of organoids derived from donated biospecimens by commercial parties (4) appreciation of donors. These themes are discussed in more detail below in a non-hierarchal manner.

Within these themes, there were no substantial differences in responses between the two groups of respondents (intestinal organoids and brain organoids). The data showed strong similarities in the themes addressed and attitudes toward the commercialization of organoids across all focus groups. One noticeable difference was that individuals with Parkinson’s and Huntington’s felt a strong urgency for applications of organoids in speeding up drug development, given the lack of reliable treatments for these diseases. This led us to believe that they were more willing to accept the potential consequences of commercialization as long as it accelerated the process of gaining access to effective drugs.

### Theme 1: benefits and concerns regarding commercial involvement

When discussing the possibility of commercialization, respondents across all focus groups reflected on the benefits and concerns of donating their biospecimen for organoid development. While respondents described commercial parties as crucial to the advancement of medical research, they also voiced concerns about excessive profits and lack of transparency, as we explain in more detail below.

#### Benefits

Respondents considered commercial parties as an integral part of the developmental pipeline of novel therapies (Table 2, quote 1). They considered that these parties provide (financial) resources and expertise to drive drug development and expand treatment options for patients (Table 2, quote 2). Specifically for participants in the focus group with Parkinson’s and Huntington’s disease, speeding up drug development was highlighted as an important benefit of involvement of commercial parties. Over all groups respondents argued that it was justified for companies to make profit given their investments and having carried the (financial) risks during drug development.

#### Concerns

Respondents also expressed concerns about the risks of commercialization. They were concerned that commercial parties could profit excessively. They feared that high profit margins could lead to disproportionate wealth for a few individuals and further increase treatment costs, which in turn could limit patient access to treatment. However, respondents struggled to define what excessive profit means to them. Some respondents expressed feeling conflicted between their desire to contribute to scientific advancements and their discomfort with supporting the significant profits of these commercial organizations.

In addition, concerns about patenting were raised, which were often seen as a way to exert power (Table 2, quote 3). Furthermore, it was deemed problematic that commercial parties hold exclusive rights to a treatment, meaning they can fix prices that far exceed actual production costs, while also keeping competitors at bay (Table 2, quote 4). One respondent pointed out that patents can lead to inefficiencies in research and scientific advancement by hindering transparency, as this requires other researchers to conduct the same research when exploring similar treatments or drugs. Respondents in all focus groups agreed that reducing monopolies in the pharmaceutical sector is important to ensure broader accessibility. However, some acknowledged that patenting is necessary for commercial parties to recoup the costs incurred during research and development.

The idea that organoids derived from their biospecimen could end up with an internationally operating or collaborating commercial party also brought up concerns. Respondents expressed unease about foreign parties having access to their biological material and personal information. This raised privacy concerns for themselves and their families (Table 2, quote 5). They worried that foreign parties can be bound to different regulatory frameworks and attain different societal values and ethical standards than in their home country (Table 2, quote 6). These uncertainties were paired with privacy and ethical oversight concerns and made respondents wary about potential misuse of their data. Respondents highlighted several examples of potential misuse. One major fear was the possibility of cloning, where copies of their DNA, organoids or even themselves entirely, might exist in multiple locations without their knowledge. Additionally, they worried that their DNA could be identified, potentially exposing genetic information about themselves and family members. Concerns also arose about what might happen if such sensitive data fell into wrong hands. Respondents feared that health insurance companies might exploit this information to increase premiums or treatment costs or that law enforcement could use it for solving crimes (Table 2, quote 7). In other cases, some even referred to the possibility of organoids being involved in organ trade. Fear of misuse of their biospecimen grew when organoids and the data are stored indefinitely. For them, this element of time increased the risk that organoids derived from their biospecimen might be used in ways that do not align with their initial intention, given the uncertainty about future applications as science continues to advance (Table 2, quote 8).

**Table 2:** Illustrative quotes about benefits and concerns regarding commercial involvement

Benefits and concerns	
*Benefits*	
1	R2.3: “Without commercial companies, the skeleton is not complete. (…) I don’t know exactly how that skeleton works, but we need commercial companies to bring something [treatment] to the market.”
2	R1.4: “The only advantage could be that a company has more financial resources. So, the studies can probably be more elaborate and include everything they want to test.”
*Concerns*	
3	R3.3: “And the pharmaceutical industry is allowed to make money from this [novel treatment] to do follow-up research or in general because they are allowed to make money. Only, it is not supposed to become some kind of tool of power or that people can make disproportional amounts of money from it, because it then indirectly feels as if you have contributed to something like that.”
4	R1.3 “(…) but you have the chance that you’re going to get a monopolist that outbids the entire market. And that the initial idea of the organoid research is no longer in mind, namely that we all work and think together to try to come to a solution. If we’re unlucky, we’ll get one bastion, one monopolist that runs off with it. That doesn’t seem to have a positive effect on the price in the long run.”
5	R2.4: “(…) it’s all about DNA. If you just run that organoid through a DNA scanner or throw it into a DNA database, you can retrieve a lot (…). And the question that I always miss, but that is a bit difficult in this scenario, is: what do you reveal about your own children. You pass on your DNA, so you are not making that choice only for yourself, but also for your own direct family.”
6	R2.1: “ (…) if it’s in another country, there will also be different legislation etc. So that’s why it feels even more unsafe if it’s in a country where people view these things differently.”
7	R2.4: “Look, the worst-case scenario, which never turns out as bad as you imagined it in practice, is what if such a database gets leaked? Then a health insurer could use it, discover all sorts of unnoticed diseases, raise premiums, etc. And something that I tend to worry a little less about is that the police might start using it for cold-case investigations. So, the fact that it’s stored somewhere doesn’t mean it will only be used for medical research. You never know what the future holds and who will gain access to it.”
8	R4.3: “Already in the 70s and 80s people knew that things would go in this direction and there were discussions about stem cells (…) at that time they said: donating stem cells is out of the question. That was not allowed. This is very different nowadays. So, I ask myself where it’s going when the state of the medical world continues to improve. At some point we could perhaps also produce people to harvest organs for example. (…) But where will we go next? Where do we draw the line? This example is unimaginable now, but it certainly will be imaginable in 50 years.”

### Theme 2: trust in involved parties in research

While discussing commercial involvement in research, respondents generally expressed distrust towards commercial parties and expressed trust in academic institutions.

#### Distrust towards commercial parties

Distrust towards commercial parties was underscored by the previously mentioned concerns about disproportional profits, limited patient access and high societal costs. In the focus groups with CF participants, this was emphasized by a historical case in which patient access to highly effective medication (Elexacaftor/Tezacaftor/Ivacaftor) for people with CF was reduced or prevented due to high drug costs. Across all focus groups, distrust was caused by the perception that commercial parties were prioritizing their benefit over the benefit of patients and society (Table 3, quote 1). For instance, they believed that commercial parties prioritized financial interests when shaping their research agendas, rather than focusing on creating societal value (Table 3, quote 2). For some respondents, these concerns led to the feeling that commercial parties would not adhere to existing agreements and regulations, potentially using organoids derived from donated biospecimens for broader purposes when these better served their interests. Some also suspected that commercial parties would lack transparency about the negative findings of their research if it would harm the companies’ interests (Table 3, quote 3). Moreover, respondents felt that the involvement of commercial parties, compared to academic parties alone, would lead to a loss of control over their donation, further reinforcing their feelings of distrust.

#### Trust in academic institutions

In contrast, respondents viewed academic institutions as trustworthy as they believed these institutions act in the best interest of patients and society (Table 3, quote 4). The non-commercial nature of these institutions reassured respondents by inducing a sense of security in their ethical practices and intentions. Respondents argued that in case of a collaboration between academic and commercial parties, an academic institution ensures ethical use of their biospecimen for organoid development. They trusted the academic institution to serve as a control mechanism over commercial parties and to uphold academic standards throughout the research process. Overall, respondents expressed a strong sense of trust in science and the scientific community (Table 3, quote 5).

#### (re)Establish trust

Respondents stressed the role of research ethics committees within academic institutions in (re)establishing trust. Respondents in all focus groups expressed faith in these committees (Table 3, quote 6). They believed these committees consisted of individuals committed to doing what is right and that they were protecting their interests by focusing on the well-being of patients and society. Some described them as independent, which made them more reliable. Importantly, they trusted the committees to exercise their authority responsibly by approving or rejecting research proposals based on ethical considerations.

Another key element affecting trust was the nature of commercial involvement. When academic partners led collaborations with commercial parties and the research was beneficial to society, respondents felt the research was more aligned with individual and social interests and retained a greater sense of control. In contrast, when a commercial party acted independently, such as by directly requesting organoids from a public biobank, trust was diminished as societal and donor interests felt less protected and control over use of organoids derived from their biospecimens was reduced. A commercial biobank elicited the most distrust, as respondents perceived it as completely undermining their confidence in prioritizing societal and individual interests and any feeling of donor control.

**Table 3:** Illustrative quotes about trust in involved parties in research

Trust	
*Distrust towards commercial parties*	
1	R2.5: “The companies may say one thing, but they mean something else. So, you can never rely on what they say. My trust in companies is very low, because their ultimate goal is to make as much profit as possible and do not care so much about the patients anymore.”
2	R4.2: “I think we expect a hospital to be more aware of the societal interests. Companies may not. Sometimes you hear that they [companies] have made a lot of progress with research, but that it is no longer financially interesting for them to continue. So, they stop and leave it, even though it would benefit a lot of people.”
3	R3.4: “ (…) I’m almost convinced that they only show the positive sides, and that the negative sides are underexposed. And that in a few years we’ll find out that there are more side effects or dangers than that were actually presented.”
*Trust in academic institutions*	
4	R2.1: “It feels like they [universities] have other interests. They are much more focused on what patients need and how they can organize that for them, while I feel that a company has other interests. Sure, they will also want to discover something or contribute to., but it’s also primarily about making money.”
5	R4.5: “My anchor point is the university. The fact that the university is in the lead in the research increases my confidence and trust in other steps. Sure, universities are also places where money needs to come in. I don’t have any illusions about that either. They also work together with companies. But in my opinion, it makes a big difference that the university as a research center is involved and stands above the company. That makes it a lot easier for me, also due to my confidence in science.”
*(re)Establish trust*	
6	R4.2: “I mean, if I didn’t trust the Medical Ethics Review Committee, I think I couldn’t trust anything at all. So, I have trust in that.”

### Theme 3: control over the use of organoids derived from donated biospecimens by commercial parties

Control was briefly mentioned in relation to trust; however, it stands out as a relevant theme on its own. Control directly influences respondent’s willingness to donate biospecimens for research with organoids and was a dominant theme in all focus groups.

#### Perceived lack of control

The need for control over how commercial parties use donated biospecimens for organoid development was a recurrent theme in all focus groups. Respondents mentioned feeling uneasy due to a perceived lack of control. Once biospecimens were handed over to commercial parties, they felt there was a lack of transparency and oversight, resulting in limited insight and influence over the current and future use of the organoids derived from their biospecimens (Table 4, quote 1–3). Respondents also argued that the potential for organoids to be multiplied, resold or repurposed without their oversight diminished their sense of control. This feeling was amplified by the increased physical distance of organoids derived from their biospecimens, as respondents associated commercial involvement with potential resale or relocation of organoids to other countries, where ethical standards may be different (Table 4, quote 4). Lack of control was a reason why some respondents would be less willing to donate or dissent altogether.

#### Promoting a sense of control

To promote a sense of control, respondents mentioned three important aspects. First, a clear desire for (international) regulations enforcing transparency and ethical use of donated biospecimens for organoid development in both current and future applications was brought up. Second, respondents expressed a desire for clear communication regarding who will make use of the organoids, for what purposes, at what location, now and in the future, along with updates on the research progress. Third, many respondents highlighted the need for an independent ethical party, free from (financial) interests in the research, to shape and enforce these regulations. They felt that this body should be responsible for determining the purpose, timeline, (international) partners, and conditions under which organoids derived from donated biospecimens can be used by commercial parties. This oversight was particularly important to the respondents if studies were exclusively conducted by commercial parties (i.e., a commercial biobank sells products to a commercial research company) without involvement of academic partners. In addition, the respondents stressed that this independent party should actively oversee adherence to the regulations. While some respondents thought this a fitting role for ethical committees associated with universities (Table 4, quote 5), others preferred an entirely independent body, free from pressure of academic achievements, to prevent conflicts of interest.

**Table 4:** Illustrative quotes about control over the use of organoids derived from donated biospecimens by commercial parties

Control	
*Perceived lack of control*	
1	R1.2: “There is less control in case of a single company. If you work together, you can still control each other. That is something that you can build-in. If a company works alone (…), there is much less control over what happens to your biopsy, the results and the follow up.”
2	R2.4: “And the fact that a hospital, an independent institution, keeps control on what happens during research. And not that a company can start research independently, like: hey, we’ve come up with something fun again, let’s see what we can do with it.”
3	R3.4: “You do lose control. Once you’ve handed it [organoid] over to a company, they can do whatever they want with it. They can do ten things and only report on one.”
4	R4.1: “The further away from the original source, the less traceable it becomes over time. You can make tons of agreements here [country of donation], but the moment the organoid is sold to ‘*Farawayistan’* where they have different rules and legislation, your oversight stops.”
*Promoting a sense of control*	
5	R4.3: “The university says it is in good hands, but I would rather have this monitored by an institution that is completely independent from companies or universities, so that there is no conflict of interest.”

### Theme 4: appreciation of donor

When discussing the willingness to donate biospecimens for organoid development for research with commercial involvement, respondents expressed the importance of feeling appreciated. They brought up several ways in which they would feel valued, including information, active engagement and rewards.

#### Informed

Many respondents mentioned that being informed about the aim, research activities and outcomes throughout the research process would make them feel valued. Respondents with prior experience in participating in studies, e.g. by donating biological material or joining an interview study, indicated that they had experienced feeling overlooked by researchers. They felt they were left in the dark about the remaining study activities and outcomes, which made them feel unappreciated and sometimes even reluctant to participate in future research (Table 5, quote 1). Some respondents suggested regular updates or an invitation to a research event throughout the process as possibilities to make them feel actively informed and respected.

#### Engaged

Some respondents also expressed a desire to be viewed as an equal partner throughout the research process. In the case of donating to a biobank, from which biospecimens can be requested for multiple studies, respondents indicated that they would like full transparency and influence for which studies their material will be used. They preferred not to donate to a ‘black box’ but rather requested active patient participation and engagement (Table 5, quote 2). Rather than performing a one-time transaction with the biobank, they wanted to be viewed as an integral partner in the research process and not be taken for granted (Table 5, quote 3, 4).

#### Rewarded

Respondents also discussed appreciation in terms of receiving benefits that recognize their contribution. Financial compensation for their tissue donation was not the primary motivation for many respondents (Table 5, quote 2, 5), although some felt it would increase their motivation (and potentially that of others) to donate biospecimens for organoid development. However, some respondents mentioned that being offered financial compensation would be counterproductive for them, as they did not want to feel like they were being bought off or have any other incentive than scientific advancement (Table 5, quote 6). When discussing commercial biobanks, which have direct financial benefits from sharing biospecimens with other parties, financial compensation became more appealing to the respondents, as they felt it fairer due to the motives of the commercial biobank. Some respondents even suggested that donors could become stakeholders in the commercial biobank or commercial party. Other forms of compensation also brought up by the respondents were receiving information on study results, discounts on future treatment or a donation to a social fund to increase patient access. One participant added that a thank you note in the form of a letter or e-mail each time the organoid was used would be well appreciated (Table 5, quote 7).

**Table 5:** Illustrative quotes about appreciation of donor

Appreciation of donor	
*Informed*	
1	R1.2: “It’s more like, I have always been very interested in everything. and now I’ve lost the connection to what they have told me and presented me at that time. Then I think: okay, I gave quite a contribution, and how are things now? I find patient participation, so involving the patients in the research — which was also mentioned — an important factor that now plays a bigger role in my consideration.
*Engaged*	
2	R1.3: “For me, compensation is not so much in materialistic things. It is rather about involvement, attention for detail and communication.”
3	R1.1: “That triggers me as a patient to ask myself: why am I not a full-fledged third partner in this? Because we need each other, that is clear.”
4	R1.1: “That I maintain grip on the study and the research process, assuming that I have sufficient knowledge to be a partner in it. And why not? (…) Even now communication and mutual information is already quite poor. That does something to my misapprehended child in psychological terms. I feel seen as lesser and taken for granted. And I wonder if that’s my problem or theirs.”
*Rewarded*	
5	R1.2:” I really don’t need a financial compensation all the time. To be involved. I would see participation as a form of compensation. That you have the feeling that you are being included.”
6	R4.5: “For me personally, receiving a [financial] compensation would mean that I would think even more critically if I want to participate in the study. It would invite me even more to find out if it is being put to good use. If I let someone pay me for something, I want it to be good even more, so it would almost work counterproductive. (…) My motivation to participate in these things would be intrinsic, namely playing a part in solving the problem. A financial compensation would problematize that.”
7	R3.2: “An email that says: thank you for participating. I don’t need them to put my name on a drug or so, maybe a bit overexaggerated. But knowing that you did something good, maybe it’s my Catholicism, is also of importance.”

## Discussion

The goal of this study was to identify and assess underlying reasons for potential donors’ reservations regarding commercial involvement in organoid research. To our knowledge, this was the first qualitative focus group study that specifically focused on the perspectives of potential donors regarding the commercialization of organoids. Commercial involvement included collaboration between academic and commercial parties or independent research by commercial parties. In the focus groups, we discussed only commercial involvement, therefore all findings refer exclusively to that context.

We hypothesized that differences in organoids would lead to variations in respondents’ answers due to differences in ethical sensitivity. However, our data did not show any substantial differences other than the strong urgency expressed by people with neurodegenerative diseases to speed up drug development and a lenience to accept commercialization as long as its related to gaining access to effective drugs. These findings must be viewed within the context of our study design, which included a specific set of participants and specific organoid models. Differences in perspectives may emerge when including different participant groups, with diverse cultural, medical, and/or socio-economic backgrounds, and when presenting them with different organoid models.

We identified four themes: While respondents recognized the benefits of commercial involvement, they also expressed concerns (theme 1). Many highlighted a lack of trust in the parties involved (theme 2) and emphasized the need for control over the use of organoids derived from donated biospecimens by commercial parties (theme 3). Additionally, respondents frequently voiced the desire for more appreciation as donors (theme 4). The themes identified in our study are applicable to broader contexts of commercialization involving human biospecimens, indicating that our findings are valuable beyond the specific context of organoids.

In what follows, we reflect on our results from which we derive recommendations for practice. We connect findings to broader literature and highlight underexplored topics. We particularly focus on what researchers and commercial parties can do as this is within the scope of our study.

### Reflection and recommendations for practice

To address the themes that were identified, we reflect on implications of our findings after which we propose several recommendations to help mitigate these issues.

#### Transparent and effective communication

In this study, we observed a lack of participants’ understanding of commercialization and who is involved in the process [23]. Some participants in our study were unaware that they had previously consented to commercialization purposes in biospecimen donation for organoid research. This confusion may stem from the vague or insufficient definitions of commercialization and commercial access in informed consent documents, which is also seen in the broader literature on biobanking documents [23]. In addition, many respondents expressed a certain fear about the commercialization of organoids, which may partly arise from lack of communication resulting in misconceptions on what is technically and/or legally possible with organoids, for example organ trade and selling personal data to insurance companies.

##### *Recommendations*

Based on this study’s findings, we can conclude that transparent and effective communication by researchers and commercial parties is crucial to help donors understand what commercialization is and to increase their willingness to donate. Therefore, we recommend that donors should receive clear information about where the organoids derived from their biospecimens are used for, where they are stored, who has access to them and what the roles and responsibilities of parties involved in the commercialization process are.

Additionally, transparent and effective communication can help to address potential donors’ concerns about commercialization. Therefore, it is important that researchers and commercial parties explain what is technically and legally possible, both now and in the future. Although it is impossible to predict future developments, researchers and commercial parties should provide follow-up communication after donation if new applications arise. Furthermore, to understand the origin of donors’ concerns with commercialization, social scientists should investigate how these concerns emerge from historical, contextual and social circumstances (including negative experiences with the healthcare system) [24].

To meet the need for transparent and effective communication of participants, researchers and commercial parties should invest in consent models that move toward dynamic consent. Dynamic consent refers to personalized, digital platforms that support both the consent process and ongoing communication between researchers and participants [25]. These platforms can accommodate various types of consent depending on the research context and objectives, and they can facilitate continuous engagement between researchers and potential donors [25].

#### Donor engagement and appreciation

Our study highlights the importance of ensuring that donors feel appreciated for their (voluntary) contributions. Our study shows that financial rewards are not seen as a primary motivation to donate biospecimens for organoid development by potential donors. Instead, efforts made by researchers to involve donors via patient participation and research updates were seen as ways to appreciate the voluntary donation of donors. However, when researchers and/or commercial parties are expected to generate profit from organoids derived from donated biospecimens, benefit-sharing becomes more important. In these instances, donors feel that the exchange is to be more transactional. This is particularly true when the organoid is not acquired by a research party directly, but instead by an intermediate party, such as a commercial biobank, where the organoid itself is sold for profit. In line with existing literature, patients increasingly appreciate compensation when their biospecimens are used for for-profit research [26]. However, a lack of clarity on the level of financial compensation remains, as respondents had difficulty specifying what they considered fair or appropriate.

##### *Recommendations*

To sustain willingness to donate in future organoid research, researchers and commercial parties should recognize donors as integral partners in the research process and demonstrate respect for their contributions. Meaningful donor engagement, such as regular research updates, personalized thank-you notes and tangible examples of scientific advancements enabled by their contributions, serves as a way to appreciate their role. This aligns with broader literature, which describes that research dissemination and participant recognition fulfill the social contract between researchers and participants, ultimately validating their participation and encouraging continued participation in future research [27, 28]. Additionally, active patient participation, in which donors are invited to provide input, helps transform the role of the donor from a passive study subject to an active (patient) partner, which gives donors a shared sense of purpose and makes them feel valued. This shift fits within a larger movement in medical research, where the role of patient participation is increasingly seen as collaborative rather than simply contributory [29, 30].

#### Oversight and legislation

Control was deemed important by potential donors when discussing the commercialization of organoids. Concerns about misuse by commercial parties were present at a national level and even more profound in the context of international collaboration. Our study indicates that potential donors value clear oversight of organoid use by commercial parties. In addition, they seek assurance that ethical standards and values of their country apply to the use of their donated biospecimens for organoid development. Many participants thought responsibility for this oversight could be appointed to ethical committees within universities. However, some participants in our study suggested that an independent party is necessary because ethical committees are still linked to the university and therefore might be biased.

##### *Recommendations*

To address concerns of potential donors, we advise researchers and commercial parties to work towards establishing regulations and mechanisms that enforce robust and transparent oversight. This requires two considerations.

First, independent parties must be appointed to ensure that oversight mechanisms are implemented by neutral organizations, minimizing the risk of conflicts of interest with both commercial and academic stakeholders. Similar to ethical committees, these independent parties should evaluate and approve the ethical integrity of research proposals involving organoids while maintaining oversight and influence over the use of donated biospecimens intended for organoid development, both now and in the future. Establishing these mechanisms at a national level helps building trust that donations are used ethically and responsibly. However, as organoid research often requires international collaboration, stressing the need for international standards. Such standards ensure consistency and transparency on how organoids are used across countries and provide assurance that the same standards apply. In addition, when international oversight differs from national standards, these limitations and challenges should be clearly communicated to the donor.

Second, researchers and commercial parties should align their practices with emerging legislation, such as the European Regulation on Substances of Human Origin (expected to come into force in 2027), which may provide additional ethical guidance on the commercialization of organoids. Meanwhile, organoid research and development continues to advance rapidly, raising questions about whether new legislation can keep pace with this progress. At the same time, legislation leaves room for interpretation. While the principle of non-commercialization of Substances of Human Origin (SoHo) is a core legal foundation of the Council of Europe, certain loopholes exist [31]. The sale of organs, tissues and blood is prohibited, yet technical services performed on these materials, such as within organoid research, may result in reasonable and proportional profit [31]. This creates a gray area, as making organoids involves processing SoHO, which can later be commercialized for profit [31]. Furthermore, the concept of ‘reasonable donor compensation’ remains undefined in both medical and research contexts, leaving it open to interpretation [31]. As a result, this loophole can be exploited to turn donations into disproportionate profits [31]. Therefore, researchers and commercial parties should seek a transparent pricing model that allows proportionate profit for commercial parties [31]. As such, further research is needed to explore what reasonable donor compensation entails and how these legislations can be effectively implemented in organoid research and treatment development with commercial parties, reducing uncertainties and increasing transparency for potential donors.

#### Reasonable trust and trustworthiness

Our study showed that (re)establishing trust is an important prerequisite for the responsible commercialization of organoid research. Within the CF focus group, issues were raised with Vertex’s Elexacaftor/Tezacaftor/Ivacaftor drug™, which has drastically improved life expectancy and lung function for people with CF but is also one of the most expensive drugs on the market [32, 33]. Therefore, global access remains limited, particularly for low-and middle-income countries [32, 34]. These high prices, which are considered unjustifiable by some experts, have resulted in significant damage to trust in pharmaceutical companies within the CF community [32, 33]. Lack of trust among potential participants towards commercial parties in research has been outlined in the international literature about biobanks and is not unique to organoids. For example, a review examining public perspectives on biobanking found that respondents from different countries, including those in North America, Africa, and Asia, consistently expressed greater trust in academic researchers compared to those funded by private-sector organizations, pharmaceutical companies or insurance providers [35].

##### *Recommendations*

(re)Establishing trust necessitates extra efforts by researchers and specifically by commercial parties. First, researchers and commercial parties should require a good understanding of what it means to be worthy of trust within a particular cultural context [36]. This includes remaining attentive to the social, political and cultural climate within which organoid research operates, and engagement with these communities could be a way to realize this [36]. Second, researchers and commercial parties should maintain trust by acting in a trustworthy manner [36]. This means for instance to transparently and comprehensively describe challenging aspects of commercialization of organoids instead of deliberately using vague language to describe commercialization in informed consent letters. Third, researchers and commercial parties must recognize that some groups have reasonable distrust due to historical exploitation in research (e.g. the case of Henrietta Lacks) or past experiences with pharmaceutical companies that set high drug prices (e.g. Vertex), making treatments unaffordable and accessible only to a select group. Re-establishing trust with groups that have been priorly harmed will demand extra efforts and safeguards, including sensitivity to past harm and community engagement [32, 33].

### Limitations

The results of this study should be interpreted within the context of several limitations. First, the majority of respondents, particularly those within the CF groups, demonstrated a strong connection to and interest in research. Many had previously donated to organoid research or were already familiar with it. This prior engagement may have introduced selection bias. It could have shaped their perspectives, potentially making them more positive or negative about organoid commercialization compared to less-informed groups. Second, the focus groups included participants who were predominantly born, raised and living in The Netherlands, reflecting mainly a Dutch perspective on the commercialization of organoids. Conducting similar studies in different global regions may yield different views and opinions on the commercialization of organoids. Third, we used vignettes to guide the focus groups. While this provided structure and clarity on the topic, it may have influenced the spontaneity of respondent’s answers and potentially steered their responses in a particular direction. To minimize this potential bias, the vignettes were reviewed by a master’s student in a pilot interview. Fourth, group dynamics in focus groups may have influenced individuals’ responses, as participants might conform to dominant opinions or participants with strong viewpoints. To minimize this effect, moderators aimed to actively engage less outspoken participants to encourage diverse perspectives. Fifth, the study is influenced by the positionality of the researchers. Different researchers might have focused on different aspects of the respondent’s answers and categorized the codes and themes differently. To mitigate this, the role of the moderator in the focus group was alternated and transcripts were coded individually by two authors with diverse backgrounds (a bioethicist and a health economist). Sixth, the sample size per focus group was rather small (*n* = 4–6), with a total sample size of *n* = 19. Qualitative studies aim for analytical generalizability and information saturation rather than statistical generalizability. In this study, key themes were consistently raised across multiple focus groups, and no new themes emerged in the last group. As such, the analysis of this study showed abstract insights concerning the ethical and economic dimensions of organoid commercialization, making the findings potentially applicable to similar contexts when examining the procedures, methodologies and analysis strategies [37]. Additionally, the analytic generalizability was strengthened by analyzing the focus groups with multiple team members and by triangulating the results with existing literature.

## Conclusion

Organoids are currently used in research and drug development and are expected to play a significant role in healthcare applications in the future. Advancing these applications requires the involvement of commercial parties and donations by humans. Even though potential donors acknowledge the benefits and need for commercial involvement to advance organoid research and drug development, many have concerns about commercial involvement. This study helps to expose and understand the concerns of potential donors. To address these concerns, we recommend that researchers and commercial parties communicate transparently and effectively, actively engage and appreciate donors, implement robust oversight mechanisms and (re)establish trust and trustworthiness through responsible practices. Further evaluation of these insights and other ethically relevant aspects of commercial involvement in organoid research should take place in co-creation with diverse stakeholders, at least including patient partners, academic and commercial researchers, biobank employees and members of the general public. In this way, these considerations can inform researchers and commercial parties, enabling them to work together toward responsible and sustainable organoid research.

## Electronic supplementary material

Below is the link to the electronic supplementary material.

Supplementary Material 1

## Data Availability

Qualitative data and vignettes are available from the corresponding author upon reasonable request.

## Abbreviations

CF: Cystic Fibrosis
SoHo: Substances of Human origin
