# Supplementary material for: The ethics and economics of organoid commercialization: potential donors’ perspectives
Source: BMC Med Ethics. 2025 Jul 29;26:109. doi: 10.1186/s12910-025-01269-3 (PMC12309163; doi:10.1186/s12910-025-01269-3)
Supplement: Supplementary file 1 — Supplementary Material 1 [file 12910_2025_1269_MOESM1_ESM.docx]

**Topic list**

Introduction

- Welcome
- Introduction researchers
- Explain purpose of the meeting:
  - We’re interested in hearing your thoughts and perspectives on the involvement of organizations such as companies and hospitals in organoid research. You don’t need any prior knowledge about organoids.
  - We’re gathering different pserpectives to support safe development and use of organoids in research, including collaborations with companies.
  - With you diagnosis, you might one day be eligible to donate tissue for this type of research. So what we’ll discuss today is hypothethical – but since you are a potential donor for organoid research, your opinion is especially valuable to us
- Emphasize that all personal data will be pseudonimyzed and encrypted.
- There are no wrong answers, we’re genuinely interested in your experiences, opinions, and perspectives.
- You may stop at any time, during or after the interview
- Ground rules:
  - What is shared in this focus group stays confidential
  - Please let others finish speaking before responding
  - You’re allowed to change your mind about any topic at any time
- Structure of the session
  - Part 1:
    - Introduction round
    - Background information
  - Part 2:
    - Scenario 1
    - Scenario 2
    - Closing discussion
- Any questions so far?
- Ask for everyone’s consent to record the session via video and audio

**PART 1**

*Introduction round*

- What is your first name?
- Have you heard of organoids before?

*Background information*

- Slide 2: What are organoids?
  Organoids are a type of 3D mini-organ, grown in a lab from donor cells. A lot of research is currently being done using organoids.
- Slide 3: Cystic fibrosis organoids resemble mini-intestines
  Researchers can grow a personalized mini-intestine from a patient's intestinal cells. Depending on the type of cell — intestinal or kidney, for example — organoids from different organs can be made. Although organoids are simplified versions, they function similarly to real organs.. This is why researchers use them as human models in their studies. For example, cystic fibrosis organoids can function like mini-intestines.
  OR
  Slide 3: brain organnoids resemble mini-brains
  Researchers can use a patient's blood cells to grow a personalized human mini-brain in the lab. Depending on the type of cell — intestinal or kidney, for example — organoids from different organs can be made. Although organoids are simplified versions, they function similarly to real organs. This is why researchers use them as human models in their studies. For example, brain organoids can function like mini-brains.
- Slide 4: How are organoids made?

Organoids are made by growing donor cells in a lab. For example, if you donate a small sample of gut tissue, researchers can extract stem cells from it. These can then be cultivated in 3D to form an organoid.

- Slide 5: What can you do with organoids?

Researchers use organoids to better understand disease and develop new medications and treatments.

- Slide 6: What is a biobank?
  Organoids from different donors are stored in a biobank, just like books in a library. Researchers can request access, but only after approval by a medical ethics committee.
- Slide 7: How is does your organoid end up in a biobank?

Researchers (for example, at UMC Utrecht) may ask to use patient tissue. A UMCU doctor may ask you if you want to donate cells. If you agree, a biopsy is taken, and organoids are created and stored indefinitely in the biobank. Researchers access them anonymously, which means that your personal data is separated from the organoid.

- Slide 8: Who can use biobanks?

Both university/academic researchers and private companies can use the biobank, and they sometimes collaborate. Today, we’ll be discussing this kind of collaboration and the sharing of organoids with companies.

- Are there any questions so far?

**PART 2:**

*Scenario 1 (slide 9)*

Base narrative: please try to imagine the scenarios as if they are about you personally.

- Slide 10: Scenario 1
  Medicijnen BV and the University Medical Center Utrecht (UMCU) want to collaborate on research to develop a medicine for cystic fibrosis/Huntington/Parkinson/epilepsy. This involves Maxime from Medicijnen BV and Udo from UMCU.
- Slide 11:
  Maxime works at Medicijnen BV, a pharmaceutical company. Udo works at UMCU and researches cystic fibrosis/Huntington/Parkinson/epilepsy. Working together allows them to make medicines available faster to the patients who need them.
- Slide 12:
  Maxime and Udo need organoids from people with cystic fibrosis/Huntington/Parkinson/epilepsy. UMCU asks patients if they’re willing to donate cells. Participation is voluntary and unpaid. Their research plan is approved by the ethics committee.
- Slide 13:

The research takes place at UMCU. Organoids do not leave the institution. Basic data (like age, gender, condition) are shared with the company in anonymized form. The results are published in an open-access journal.

- Slide 14:

Based on the findings, Medicijnen BV develops a successful medicine and brings it to market, making a profit.

- Slide 15:

Your organoids are kept in the biobank for indefinite future use.

| **Discussion questions** | **Underlying questions** |
| --- | --- |
| What is your initial thought? | Emotions, thoughts, what do you feel/think? |
| Do you find this scenario acceptable? Why or why not? | What role does the company play? Is innovation in medicine possible without such partnerships? |
| Do you see any risks or benefits in this scenario? Any concerns? | E.g. indefinite storage, profit margins, scientific accessibility, inequality in healthcare |
| Would you be willing to donate your cells in this scenario? Why or why not? |  |

Now we are going to adjust the scenario at certain levels and see what your opinion is. This will happen on three levels: collaboration, compensation and outcomes (slide 16).

Collaboration

- Slide 17:
  Maxime wants to conduct research on medicines for cystic fibrosis/Huntington/Parkinson/epilepsy using organoids
- Slide 18: Maxime collects organoids for her research (click animation)
  In the previous scenario, Maxime from Medicijnen BV and Udo from UMC Utrecht worked together. Imagine that Maxime is now no longer collaborating with Udo. Maxime decides to independently request tissue samples from the UMC Utrecht biobank. For this, approval from the medical ethics committee is again required. Medicijnen BV pays a certain amount to the UMC Utrecht biobank. The organoids are then sent to Medicijnen BV. Basic data such as your gender, age, and disease are shared with the company, but anonymized before being shared.
- **Slide 19: Maxime conducts the study and publishes the results**
  The research is carried out by Maxime at Medicijnen BV. After two years, the study is completed and your organoids are returned to the UMC Utrecht biobank. The research findings are published so that everyone can read them.
- Slide 20: **Maxime’s company develops a medicine and brings it to market**
  A few years later, Medicijnen BV is able to bring the medicine to market. The medicine works well and can be prescribed to patients. Medicijnen BV makes a profit from the medicine.

| **Discussion questions** | **Underlying questions** |
| --- | --- |
| What is your initial thought? | Emotions, thoughts, what do you feel/think? Moral intuitions |
| Do you find this scenario acceptable? Why or why not? | A medical ethical review committee application is still required. What conditions need to be in place for it to be acceptable? |
| Do you see any risks of benefits in this scenario? Any concerns? | No longer involvement from an academic institution |
| Would you be willing to donate your cells in this scenario? Why or why not? | Conditions for donation Would this senceario reduce willingness to donate among potential donors? Why or why not? |

Compensation (slide 21)

Slide 22: Currenlty, you receive no compensation. Imagine you do receive compensation. What are your thoughts?

| **Discussion questions** | **Underlying questions** |
| --- | --- |
| What is your initial thought? | Emotions, thoughts, what do you feel/think? Moral intuitions Why would you want or not want compensation? |
| What should that compensation consist of? | Should it be financial compensation? What amount should be appropriate? Could there be other forms of compensation? Fo rexample, would early access to medicines be an acceptable form of compensation? |
| Do you find this scenario acceptable? Why or why not? | Is this scenario fairer considering the profit that Medicijnen BV will make? |
| Do you see any risks of benefits in this scenario? Any concerns? | Would you be more inclined to donate because financial compensation is involved? Bias?  Should donation be altruistic? Why? |
| Would you be willing to donate your cells in this scenario? Why or why not? | Conditions for donation Would this senceario reduce willingness to donate among potential donors? Why or why not? |

Outcome changes (slide 23)

- Slide 24**: Maxime conducts the research and keeps the results to herself**
  In the current scenario, profit is made and the research is published so that everyone can read the results. But what if the results are only accessible to Medicijnen BV?

| **Discussion questions** | **Underlying questions** |
| --- | --- |
| What is your initial thought? | Emotions, thoughts, what do you feel/think? Moral intuitions |
| Do you find this scenario acceptable? Why or why not? | Medicijnen BV has an advantage over other pharmaceutical companies, may apply for patents, and has an incentive to invest the medicine. |
| Do you see any risks of benefits in this scenario? Any concerns? | Will a medicine still reach the market this way? Redcued competition possible? Not accessible to the public |
| Would you be willing to donate your cells in this scenario? Why or why not? | Conditions for donation Would this senceario reduce willingness to donate among potential donors? Why or why not? |

*Scenario 2*

- Slide 25: **Scenario 2. Medicijnen BV buys tissues via the internet**
  Forget the previous scenario. We are now starting a new narrative.
- Slide 26: Maxime wants to research medicines for cystic fibrosis/Huntington/Parkinson/epilepsy using organoids

Medicijnen BV wants to develop a medicine for people with cystic fibrosis/Huntington/Parkinson/epilepsy. Maxime works at Medicijnen BV, a company that aims to develop and sell medicines.

- Slide 27: **Maxime buys organoids from a biotech company (BioBV)**
  To research the new medicine, Maxime needs organoids from people with cystic fibrosis/Huntington/Parkinson/epilepsy. Maxime purchases these organoids online from BioBV, a well-known biotechnology company.
- Slide 28: **BioBV collects organoids and sells them to companies like Medicijnen BV**
  BioBV has asked donors, including you, for permission to collect and sell these organoids. Maxime receives not only the organoids, but also anonymized basic information about the donors, such as gender, age, and disease, along with proof of the donor’s consent.
- Slide 29: **Maxime conducts the research**The research is carried out at Medicijnen BV.
- **Slide 30: Maxime’s company develops a medicine and brings it to market**Based on the results, a new medicine is developed. The medicine turns out to be effective for people with cystic fibrosis/Huntington/Parkinson/epilepsy. It is approved and brought to market. Medicijnen BV makes a profit from the medicine.
- **Slide 31: The organoids are stored at Medicijnen BV**
  Once the research is completed, the organoids are stored at Medicijnen BV for potential future studies. Other researchers within Medicijnen BV may also use the organoids for different studies.

| **Discussion questions** | **Underlying questions** |
| --- | --- |
| What is your initial thought? | Emotions, thoughts, what do you feel/think? Moral intuitions |
| Do you find this scenario acceptable? Why or why not? | Faster innovation |
| Do you see any risks of benefits in this scenario? Any concerns? | What happens to trust if there is no longer an academic partner involved? Who owns the bodily material? Who is responsible for safety and minimizing risks?  Privacy concerns |
| Would you be willing to donate your cells to BioBV in this scenario? Why or why not? | Conditions for donation Would this senceario reduce willingness to donate among potential donors? Why or why not? Compensation  What is (un)acceptable? |

Outcomes (slide 32)

- **Slide 33: Your donated organoids are sold by BioBV to other parties, such as companies and scientists**
  Imagine you decide to donate your organoids to BioBV. BioBV then sells your organoid. It turns out that your organoid has perfect properties for research. This makes your organoid attractive to scientists and other parties around the world, leading to it being sold and replicated many times. BioBV makes a profit from this.

| **Discussion questions** | **Underlying questions** |
| --- | --- |
| What is your initial thought? | Emotions, thoughts, what do you feel/think? Moral intuitions |
| Do you find this scenario acceptable? Why or why not? | The field of research is being advanced |
| Do you see any risks of benefits in this scenario? Any concerns? | Commodification/exploitation of the body What would be a fair compensation for the individual? What are BioBV’s responsibilities? |
| Would you be willing to donate your cells to BioBV in this scenario? Why or why not? | Conditions for donation Would this senceario reduce willingness to donate among potential donors? Why or why not? Forms of consent |

*Closing discussion*

**Are there any final topics we should discuss that haven’t yet come up?**
If not, we would like to thank you very much for your participation.
